# Serotonin, Amygdala and Fear: Assembling the Puzzle

**DOI:** 10.3389/fncir.2016.00024

**Published:** 2016-04-05

**Authors:** Marco Bocchio, Stephen B. McHugh, David M. Bannerman, Trevor Sharp, Marco Capogna

**Affiliations:** ^1^MRC Brain Network Dynamics Unit, Department of Pharmacology, University of OxfordOxford, UK; ^2^Department of Experimental Psychology, University of OxfordOxford, UK; ^3^Department of Pharmacology, University of OxfordOxford, UK

**Keywords:** serotonin transporter gene, serotonin, mouse models, amygdala, interneurons, SSRI antidepressants, fear

## Abstract

The fear circuitry orchestrates defense mechanisms in response to environmental threats. This circuitry is evolutionarily crucial for survival, but its dysregulation is thought to play a major role in the pathophysiology of psychiatric conditions in humans. The amygdala is a key player in the processing of fear. This brain area is prominently modulated by the neurotransmitter serotonin (5-hydroxytryptamine, 5-HT). The 5-HT input to the amygdala has drawn particular interest because genetic and pharmacological alterations of the 5-HT transporter (5-HTT) affect amygdala activation in response to emotional stimuli. Nonetheless, the impact of 5-HT on fear processing remains poorly understood.The aim of this review is to elucidate the physiological role of 5-HT in fear learning via its action on the neuronal circuits of the amygdala. Since 5-HT release increases in the basolateral amygdala (BLA) during both fear memory acquisition and expression, we examine whether and how 5-HT neurons encode aversive stimuli and aversive cues. Next, we describe pharmacological and genetic alterations of 5-HT neurotransmission that, in both rodents and humans, lead to altered fear learning. To explore the mechanisms through which 5-HT could modulate conditioned fear, we focus on the rodent BLA. We propose that a circuit-based approach taking into account the localization of specific 5-HT receptors on neurochemically-defined neurons in the BLA may be essential to decipher the role of 5-HT in emotional behavior. In keeping with a 5-HT control of fear learning, we review electrophysiological data suggesting that 5-HT regulates synaptic plasticity, spike synchrony and theta oscillations in the BLA via actions on different subcellular compartments of principal neurons and distinct GABAergic interneuron populations. Finally, we discuss how recently developed optogenetic tools combined with electrophysiological recordings and behavior could progress the knowledge of the mechanisms underlying 5-HT modulation of fear learning via action on amygdala circuits. Such advancement could pave the way for a deeper understanding of 5-HT in emotional behavior in both health and disease.

## Introduction

The amygdala, an almond-shaped structure in the medial temporal lobe, is thought to be critical for emotional processing (Klüver and Bucy, 1938; Weiskrantz, 1956; LeDoux, 2000). In recent years, the dissection of the amygdala microcircuits controlling conditioned fear has provided fundamental insights into the neurobiology of emotion (for review, see Duvarci and Pare, 2014; Tovote et al., 2015). Dysregulation of amygdala circuitry is thought to contribute to symptoms in many psychiatric disorders including depression (Sheline et al., 2001; Victor et al., 2010), post-traumatic stress disorder (Rauch et al., 2000; Protopopescu et al., 2005), social phobia (Tillfors et al., 2001; Furmark et al., 2002) and other phobias (Ahs et al., 2009). Therefore, understanding amygdala circuitry may ultimately lead to improved therapies for psychiatric disorders.

In mammals, the amygdala is densely innervated by fibers releasing 5-hydroxytryptamine (5-HT), which arise from the midbrain raphe nuclei (Parent et al., 1981). The 5-HT system appears to be crucial for myriad brain functions, including sleep, appetite, sensory processing, motor activity, cognition and emotion. In particular, the 5-HT system has long been implicated in the regulation of aversive emotions such as fear and anxiety (Deakin and Graeff, 1991; Lowry et al., 2005). Two aspects suggest that 5-HT may influence emotional processing, at least in part, via modulation of amygdala function. First, drugs that block the re-uptake of 5-HT (e.g., selective serotonin reuptake inhibitors, SSRIs), the first-line treatment for depression and anxiety (Preskorn et al., 2004), affect amygdala activation to emotional stimuli (Bigos et al., 2008; Murphy et al., 2009; Godlewska et al., 2012). Second, genetic variations in the 5-HT transporter (5-HTT) influence amygdala activation to aversive stimuli, as well as expression of anxiety-related personality traits and risk for affective disorders (Lesch et al., 1996; Hariri et al., 2002).

Notably, not all studies are consistent with the involvement of human 5-HTT gene variations in fear memory processing (Murphy et al., 2013), which has cast doubt on the link between 5-HT neurotransmission, amygdala and fear. However, human findings are complicated by environmental and demographic factors. In addition, the techniques available to study human brain function, such as non-invasive functional neuroimaging, lack the necessary spatial and temporal resolution to reveal how 5-HT impacts upon specific amygdala microcircuits. Here we review molecular, anatomical, electrophysiological and behavioral data to elucidate the physiological role of 5-HT on amygdala function and fear learning. We focus on the rodent basolateral amygdala (BLA) because this nucleus receives a dense projection from 5-HT neurons of the dorsal raphe nuclei (DRN) and is well understood functionally. We propose that physiologically released 5-HT in the BLA shapes fear learning via action on defined cell types and control of synaptic plasticity.

## Do DRN 5-HT Neurons Encode Aversive Signals?

To understand the role of 5-HT in fear learning, it is crucial to determine whether aversive stimuli and aversive cues lead to a modulation of 5-HT neurons’ firing and, as a result, to an alteration of 5-HT levels in the amygdala. The main source of 5-HT modulation of the amygdala is the DRN in the midbrain. Although it is not clear whether DRN neurons release 5-HT even at low firing rates or only above a certain threshold, it is generally believed that increased firing rates of DRN 5-HT neurons translate into elevated 5-HT levels in target forebrain areas, including the BLA. In line with this assumption, the firing rates of DRN 5-HT neurons and forebrain 5-HT concentrations are high during wakefulness and low during sleep (Portas et al., 1998; Sakai, 2011).

In the laboratory, fear learning can be assessed with a high level of control through Pavlovian fear conditioning, which involves pairing a conditioned stimulus (CS; e.g., an auditory tone) with an unconditioned stimulus (US; e.g., an electric shock). The *acquisition* of an associative fear memory during this *training* session causes the presentation of the CS only during the *test* (or *fear retrieval*) session to elicit fear responses. Although 5-HT neurons have been hypothesized to encode aversive cues and to generate aversive prediction error signals (Deakin and Graeff, 1991; Daw et al., 2002; Dayan and Huys, 2009), recordings from identified 5-HT neurons during fear conditioning have not been reported.

Nonetheless, some DRN 5-HT neurons display phasic increases in firing rates at the onset of footshocks in anesthetized rats (Schweimer and Ungless, 2010) and following punishment (air-puff to the eye) in awake, head-fixed mice (Cohen et al., 2015). In keeping with this, an increased c-Fos expression in DRN 5-HT neurons was observed following presentation of noxious stimuli (Grahn et al., 1999; Takase et al., 2004, 2005). Conversely, imaging experiments from the DRN of freely moving mice have shown that 5-HT cells do not display significant calcium transients following footshock presentations (Li et al., 2016).

In addition to noxious stimuli *per se*, presentation of a CS previously paired with a shock has also been shown to promote c-Fos expression in DRN 5-HT cells (Spannuth et al., 2011). However, Cohen et al. (2015) found that an odor CS that predicted punishment did not produce phasic excitation of 5-HT neurons in head-fixed mice. Future investigations are needed to comprehend the discrepancies between these studies. Since the DRN 5-HT neurons are topographically organized according to the areas they innervate (Jacobs et al., 1978; Imai et al., 1986), some inconsistency might reside in the portion of the DRN where neurons were recorded or imaged.

Despite these controversies, microdialysis studies suggest that both CS and US presentations are capable of enhancing 5-HT release in the BLA, with increased 5-HT in response to inescapable shocks (Amat et al., 1998), psychological stress (Kawahara et al., 1993) and fear memory retrieval (Zanoveli et al., 2009). Interestingly, these rises in 5-HT levels are slow and long-lasting (peaking at ~30 min from CS/US presentation; Yokoyama et al., 2005; Zanoveli et al., 2009). Hence, BLA-projecting DRN 5-HT neurons might signal aversion not via phasic increases in firing rate upon CS/US presentation, but rather by progressive enhancement in their frequency of discharge.

In summary, there is evidence that DRN 5-HT neurons modulate BLA circuits during fear conditioning by enhancing the release of 5-HT. However, there is mixed evidence about the phasic activation of 5-HT neurons by unconditioned and conditioned aversive cues. Recordings from identified DRN 5-HT neurons during different phases of fear conditioning are needed to elucidate their physiology in this paradigm. Additionally, the relationship between DRN neurons’ firing rates and BLA 5-HT release should be directly investigated.

## Impact of Genetic Variations of the 5-HTT on the BLA and Fear Learning

After reviewing the evidence that 5-HT levels increase in the BLA following aversive stimuli, we now ask whether genetic variation in the 5-HT system affects fear learning. The neurotransmission of 5-HT is tightly regulated by the 5-HTT, a transmembrane protein that clears 5-HT from the extracellular space and therefore controls the duration and extent of 5-HT neurotransmission (Blakely et al., 1994; Torres and Amara, 2007). In humans, a 44 base pair insertion/deletion polymorphism in the 5-HTT gene promoter region (5-HTT gene linked polymorphic region or *5-HTTLPR*) gives rise to long (*L*) and short (*S*) allele variants. The S allele is associated with reduced transcriptional efficacy, and hence lower transporter expression, leading to reduced 5-HT re-uptake and high extracellular 5-HT levels (Greenberg et al., 1999). This variation has been associated with greater risk for anxiety-related traits (Lesch et al., 1996) and post-traumatic stress disorder (Lee et al., 2005). Furthermore, *S* carriers show heightened fear learning (Garpenstrand et al., 2001; Brocke et al., 2006; Lonsdorf et al., 2009) and increased depression/anxiety susceptibility (Lesch et al., 1996), particularly when combined with adverse environmental factors (Caspi et al., 2003; Uher and McGuffin, 2010). Notably, the *5-HTTLPR* is also associated with functional alterations in amygdala activity. Specifically, compared to *LL* homozygotes, *S* carriers exhibit greater amygdala activation to fearful faces (Hariri et al., 2002, 2005) and reduced amygdala-medial prefrontal cortex (mPFC) connectivity (Canli et al., 2005; Pezawas et al., 2005). However, the association between *5-HTTLPR* genotype and amygdala reactivity in humans remains controversial and the effect size is small (Murphy et al., 2013).

**Figure 1:**
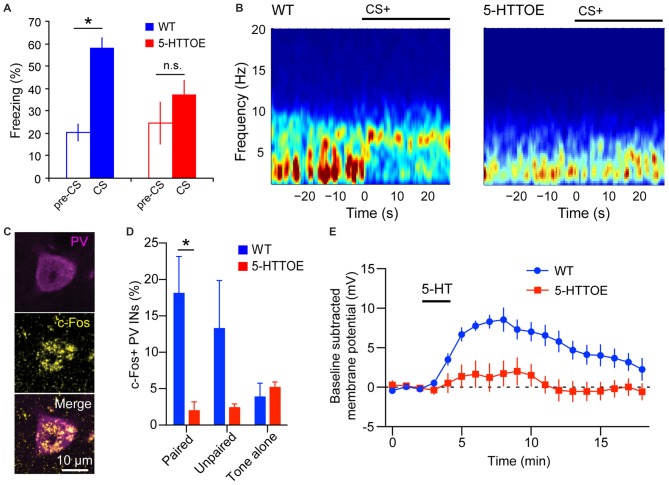
**Reduced basolateral amygdala (BLA) theta oscillations and recruitment of parvalbumin-expressing (PV) Interneurons (INs) in 5-hydroxytryptamine transporter over expressing (5-HTTOE) mice. (A)** Wild-type mice (WT) exhibit significantly increased freezing during conditioned auditory tone conditioned stimulus (CS), whereas 5-HTTOE mice do not. **(B)** Representative spectrograms showing auditory cue-evoked oscillations in the BLA of a WT and 5-HTTOE mouse. CS+ presentation evokes a higher increase in oscillations in the theta band (5–12 Hz) in the BLA of the WT compared to the 5-HTTOE mouse. **(C)** Representative BLA PV+ neuron from a WT mouse that was activated by fear memory retrieval (c-Fos immunopositive). **(D)** BLA PV INs of WT mice are activated significantly more by fear memory retrieval than PV INs of 5-HTTOE mice. **(E)** BLA PV INs of WT mice display a much stronger depolarization than 5-HTTOE PV INs when 5-HT (50 μM) is bath applied. *p < 0.05. **(A,B)** Adapted from Barkus et al. (2014). **(C–E)** Adapted from Bocchio et al. (2015).

Mice with genetically modified 5-HTT expression offer a more controlled model to investigate the impact of 5-HTT variation on fear learning and amygdala function. 5-HTT knock-out (5-HTTKO) mice display higher extracellular 5-HT levels (Mathews et al., 2004; Jennings et al., 2010) and impaired recall of fear extinction compared to wild-type littermate controls (Wellman et al., 2007). Furthermore, 5-HTTKO mice exhibit abnormal dendritic spine density of BLA principal neurons (PNs) (Wellman et al., 2007). Conversely, 5-HTT overexpressing (5-HTTOE) mice have lower extracellular 5-HT levels than WT littermate controls (Jennings et al., 2006, 2010) and exhibit impaired fear learning (Barkus et al., 2014; Line et al., 2014; Bocchio et al., 2015; McHugh et al., 2015; Figure 1A). Collectively, these findings support a positive correlation between 5-HT levels and fear learning, potentially, at least in part, via the action of physiologically released 5-HT on BLA circuits.

However, it should be kept in mind that constitutive genetic alteration of 5-HTT expression will likely change 5-HT signaling during brain development. It is therefore unclear whether effects on fear learning originate from: (1) 5-HT neurotransmission in adulthood; or (2) altered neuronal circuit development; or both of these factors.

Supporting the developmental account, several lines of evidence suggest that life-long changes in 5-HTT expression result in compensatory changes in 5-HT receptor expression and function in rodents. Namely, altered 5-HT1A (Holmes et al., 2003) and 5-HT2A (Li et al., 2003; Jennings et al., 2008; Bocchio et al., 2015) functions have been reported in both 5-HTTKO and 5-HTTOE mice. Furthermore, interfering with 5-HT neurotransmission during development alters emotional behavior in adulthood (Gross et al., 2002; Ansorge et al., 2004). Thus, studies with temporally precise and reversible manipulations of 5-HT neurotransmission are imperative to verify that 5-HT levels at the time of behavioral testing are responsible for the effects on fear learning.

### Impact of 5-HT Neurotransmission Manipulation on Fear Learning

One approach to confirm the causal link between high extracellular 5-HT levels and fear learning is the pharmacological blockade of the 5-HTT with SSRIs. This intervention raises extracellular 5-HT levels reversibly, in principle conferring higher temporal resolution than genetic variations to evaluate changes in behavior. Notably, acute and chronic administration of SSRIs appears to produce different effects.

Acute citalopram treatment increases 5-HT levels in the amygdala (Bosker et al., 2001). In humans, citalopram administration (20 mg) 2–3 h before acquisition increases fear-potentiated startle (Browning et al., 2007; Grillon et al., 2007). In rats, acute citalopram (intraperitoneal injection, 10 mg/kg, equivalent to 25 mg in humans according to plasma drug levels) before either auditory fear conditioning or fear expression increases freezing responses (Burghardt et al., 2004, 2007). Together, these data suggest that transitory increases in extracellular 5-HT levels facilitate both the acquisition and the expression of conditioned fear in a cued fear conditioning paradigm. In contrast, chronic citalopram administration in humans (10 mg/day for 2 days followed by 20 mg/day for 12 days) does not affect fear-potentiated startle (Grillon et al., 2009). In rats, chronic citalopram administration (10 mg/kg for 21 days) before (and during) fear acquisition decreases freezing during fear expression (Burghardt et al., 2004). This suggests that acute and chronic SSRI treatments have markedly different effects on fear learning, in agreement with their dichotomous effects on emotionality (acute treatment increases anxiety, whereas chronic administration has stronger antidepressant actions). Many attempts have been made to explain SSRI acute and chronic effects on the healthy and pathological brain (e.g., Harmer and Cowen, 2013), but a conclusive account is still lacking. The discrepant action on fear memory retrieval could be partially explained by the fact that chronic SSRI treatment causes whole brain adaptive to changes to 5-HT receptors (Klimek et al., 1994; El Mansari et al., 2005), as well as downregulation of the NR2B subunit of NMDA receptors in the BLA (Burghardt et al., 2004). More studies are needed to provide a mechanistic understanding of the impact of short-term vs. long-term changes in 5-HTT function.

Whole brain 5-HT levels can be manipulated also through dietary depletion of the 5-HT precursor tryptophan. In humans, this treatment decreases the recognition of fearful faces (Harmer et al., 2003) and reduces autonomic responses to cues indicating imminent aversive stimuli (Hindi Attar et al., 2012). Thus, based on SSRI administration and dietary tryptophan depletion studies, 5-HT levels seem to contribute to fear memory acquisition and expression. This contribution is likely to occur, at least in part, via modulation of amygdala circuits, because the amygdala is a crucial structure for the encoding of cued conditioned fear. Nevertheless, SSRI treatment results in changes in 5-HT levels in the whole brain. This indicates that SSRIs might modulate fear learning not only via altered 5-HT neurotransmission in the BLA, but also via concomitant action on other brain regions involved in defensive behavior, such as the hippocampus (HPC), the medial prefrontal cortex (mPFC) and the periaqueductal gray.

To confirm the impact of 5-HT on the amygdala circuits, selective lesions of 5-HT terminals can be performed in rodents using the neurotoxin 5,7-dihydroxytryptamine (5,7-DHT; Baumgarten and Björklund, 1976). Notably, lesion of 5-HT fibers in the BLA reduces freezing to both tone CSs (during acquisition and retrieval) and the training context (during retrieval; Izumi et al., 2012; Johnson et al., 2015). However, one study has reported enhanced fear-potentiated startle in rats with 5,7-DHT lesions restricted to the lateral amygdala (LA; Tran et al., 2013).

Manipulation of endogenous 5-HT release can now be achieved in rodents with high spatial and temporal selectivity using optogenetics. Recently, Baratta et al. (2015) have shown that optical inhibition of DRN 5-HT axon terminals in the BLA during CS-US pairings (or unpaired USs) during the acquisition phase impaired fear memory retrieval. However, this deficit occurred only when mice were subjected to 2 days of stress prior to fear conditioning, but not in unstressed mice.

Taken together, the data presented in this section suggest that 5-HT neurotransmission in the BLA is involved in the acquisition (and probably the expression) of cued conditioned fear. It remains to be confirmed whether the optogenetic activation of DRN 5-HT axons in the BLA could alter fear memory acquisition and expression, thereby demonstrating not only the necessity but also the sufficiency of BLA 5-HT. Undoubtedly, the link between 5-HT-dependent stress and fear learning also deserves further attention.

## BLA Circuit Dynamics Underlying Fear Learning

To understand how BLA 5-HT neurotransmission influences fear learning, it is important to use circuit-based approaches and determine how 5-HT modulates BLA microcircuits. In the last decades, research in rodents has provided unprecedented knowledge about the neurobiological mechanisms underlying fear conditioning.

A simple circuit model of fear learning (Sigurdsson et al., 2007) suggests that information about tone and shock is relayed, via glutamatergic fibers, from sensory areas of thalamus and cortex to PNs lateral (LA) subdivision of the BLA. Prior to fear conditioning these inputs are weak, but pairing the tone and shock together strengthens CS-specific inputs to specific ensembles of PNs via long-term potentiation (LTP; Romanski et al., 1993; McKernan and Shinnick-Gallagher, 1997; Rogan et al., 1997; Goosens et al., 2003; Collins and Paré, 2000; Nabavi et al., 2014). The LA projects to the basal (BA) subdivision of the BLA, and both LA and BA project to the central nucleus of the amygdala (CeA), in addition to other brain regions. The CeA contains mostly GABAergic neurons that innervate several brain stem nuclei to promote defensive responses (Veening et al., 1984; Ciocchi et al., 2010; Penzo et al., 2014). For example, CeA projections to the nucleus reticularis pontis caudalis mediate acoustic startle reflexes (Davis et al., 1982), and CeA projections to the periaqueductal gray region mediate freezing responses (LeDoux et al., 1988; Amorapanth et al., 1999; Penzo et al., 2014). Thus, LTP at synapses between CS-relaying axons and LA PNs following fear conditioning is thought to contribute to freezing responses at subsequent CS presentations via action of BLA PNs on CeA neurons.

This circuit model places significant emphasis on glutamatergic BLA PNs, pyramidal-like projection neurons with spiny dendrites that account for ~80% of BLA cells (McDonald, 1982) and which occupy a key position between sensory input and behavioral output. However, it is important to keep in mind that their activity is tightly coordinated by the release of GABA from BLA interneurons (INs), which are characterized by mostly aspiny dendrites and widely branching local axons (McDonald, 1982; Millhouse and DeOlmos, 1983). BLA INs provide feedforward inhibition to LA PNs and act as gatekeepers of synaptic plasticity because depression of feedforward inhibition is necessary to induce LTP at BLA PNs (Watanabe et al., 1995; Bissière et al., 2003; Tully et al., 2007; Morozov et al., 2011).

**Figure 2:**
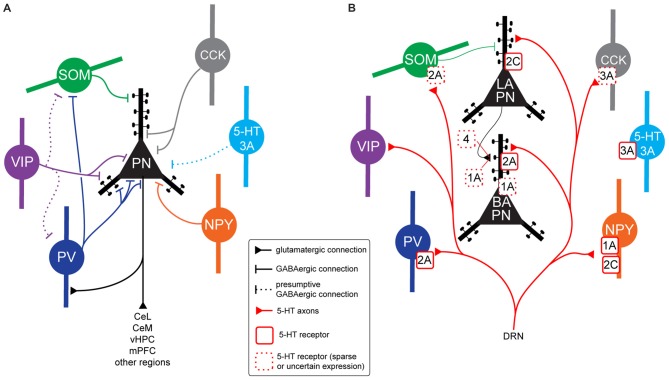
**BLA neurons connectivity, 5-HT innervation and 5-HT receptor expression. (A)** Connectivity between BLA principal neurons (PNs) and interneuron (IN) populations in rodents. Parvalbumin (PV) INs target the perisomatic region of PNs (soma, proximal dendrites, axon initial segment), but also inhibit somatostatin-expressing (SOM) INs, disinhibiting PNs distal dendrites. Vasoactive intestinal peptide (VIP) INs inhibit PN soma and proximal dendrites. As they have been shown to inhibit high numbers of Calbindin (CB)-expressing (CB+) INs, and the majority of CB+ INs are either PV or SOM, VIP INs could target PV and/or SOM INs, disinhibiting specific PN subcellular compartments, but this has not been demonstrated. The VIP-negative CB1R+ population of Cholecystokinin (CCK) INs (large CCK) target PN perisomatic region. Although it is likely that CCK-negative 5-HT3A+ INs contact PNs, this has not been shown experimentally. A subset of neuropeptide Y (NPY) INs (neurogliaform cells) have been shown to inhibit PN soma and proximal dendrites. **(B)** Expression of 5-HT receptors in neuron types of the BLA in rodents. All the IN populations illustrated have been shown to receive somatic and/or dendritic 5-HT innervation (apart from CCK-negative 5-HT3A INs). PV INs express excitatory, metabotropic 5-HT2A receptors. Thirty percent of SOM INs also seem to express 5-HT2A. Ionotropic, excitatory 5-HT3A receptors are expressed by a small proportion of CCK INs and a population of INs not expressing any other known marker. NPY INs express either inhibitory, metabotropic 5-HT1A receptors or excitatory, metabotropic 5-HT2C receptors. In some cases, both receptors could be localized on the same neuron. It is not clear what 5-HT receptor may be expressed by VIP INs. In the whole BLA, PNs are innervated by 5-HT axons only on dendrites, often on spines. In the lateral amygdala (LA), they appear to express excitatory 5-HT2C receptors mediating membrane depolarization. Axon terminals of LA PNs innervating basal (BA) PNs might express 5-HT1A and/or 5-HT4 receptors. In the BA, PNs seem to express 5-HT2A receptors on their distal dendrites. These receptors do not lead to depolarization, they might rather favor synaptic plasticity of excitatory inputs. A proportion of BA PNs might also express inhibitory 5-HT1A receptors, mediating membrane hyperpolarization. Abbreviations: DRN, dorsal raphe nuclei.

BLA INs are heterogenous and can be classified according to their neurochemical profile, and in particular the expression of specific calcium binding proteins or neuropeptides (Table 1, Figure 2A; for review Capogna, 2014). Defined IN types control the inhibition of BLA PNs subcellular compartments, altering PN integration of excitatory inputs and modulating PN spike timing. For example, during US presentation, both parvalbumin-expressing (PV) and somatostatin-expressing (SOM) INs are inhibited, leading to robust somatic and dendritic disinhibition of PNs (Wolff et al., 2014). In contrast, presentation of the CS produces excitation of PV INs, which in turn inhibit SOM INs, leading to disinhibition on PN dendrites, with a facilitation of excitatory input integration by PNs (Wolff et al., 2014).

**Table 1:** **Features and 5-HT receptor expression of main BLA GABAergic classes**.

Marker	% of BLA INs	Known cell types	Known postsynaptic targets in BLA	Activity during fear conditioning	5-HT receptor expression	Functional effects of 5-HT
PV (high overlap with CB)	~50%	Basket; Axo-axonic; AStria projecting	PN; PV; SOM (perisomatic region)	Inhibited during US, excited during CS (on average)	5-HT2A	Slow depolarization
SOM (high overlap with CB)	11–18%	Dendrite-projecting INs; Long-range projection neurons	PN (mostly distal dendrites, few proximal dendrites and somata); PV; VIP; SOM	Inhibited during US and CS (on average)	5-HT2A (only 30%, to be verified)	Slow depolarization? Hyperpolarization via GABA release from PV INs?
VIP (high overlap with CR, some overlap with CCK)	~10%	Small CCK; VIP (CCK-negative)	PN (soma and distal dendrites), CB, VIP	Unknown	Unknown	Unknown
5-HT3A (CCK-negative)	6–12%	Unknown	Unknown	Unknown	5-HT3A	Fast depolarization
NPY (high overlap with CB and SOM)	3–6%	Neurogliaform; Long-range projection neurons	PN (mostly proximal dendrites, but also somata)	Unknown	5-HT1A; 5-HT2C	Slow hyperpolarization (1A) and/or slow depolarization (2C)?
CCK/CB1R (some overlap with CB)	~4%	Large CCK	PN, CCK (perisomatic region)	Unknown	5-HT3A(only 8–16%)	Fast depolarization?

*Abbreviations: CB, calbindin; CB1R, cannabinoid 1 receptor; CCK, cholecystokinin; NPY, neuropeptide Y; PN, principal neuron; PV, parvalbumin; SOM, somatostatin; VIP, vasoactive intestinal peptide; CS, conditioned stimulus; US, unconditioned stimulus*.

As discussed in the next sections, 5-HT regulates the excitability and the integration of excitatory inputs of BLA PNs, and also their inhibition by GABAergic INs. Through these actions on BLA circuits, 5-HT can shape BLA output to the CeA and ultimately, the physiological and behavioral manifestations of fear.

## 5-HT Innervation of BLA Neurons

**Figure 3:**
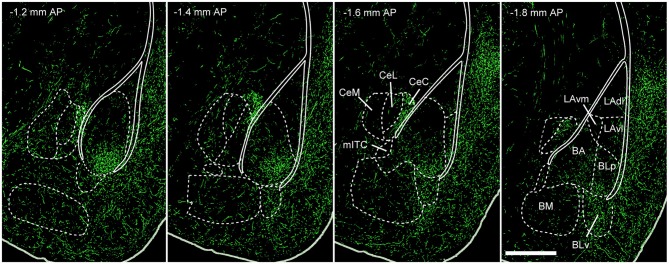
**5-HT innervation of the mouse amygdala.** Green fluorescent protein (GFP) expressed in 5-HTT + DRN neurons via injection of the Cre-dependent anterograde tracer rAAV2/1.hSynapsin.EGFP.WPRE.bGH in the DRN of SLC6A4-Cre (5-HTT-Cre) mice. This produces a pattern of innervation of the amygdaloid complex that is similar to the one previously described using antibodies for 5-HT or the 5-HTT. The densest innervation occurs in BA and BLp nuclei. Weaker innervation is observed in LA, BM and CeA, with the exception of CeC. Abbreviations: BA, basal amygdala; BLp, basolateral amygdala posterior portion; BLv, basolateral amygdala ventral portion; BM, basomedial amygdala; CeA, central nucleus of the amygdala; CeC, centrocentral amygdala; CeL, centrolateral amygdala; CeM, centromedial amygdala; LAdl, lateral amygdala dorsolater portion; LAvl, lateral amygdala ventrolateral portion; LAvm, lateral amygdala ventromedial portion; mITC, main intercalated cell cluster nucleus; AP, antero-posterior (distance from bregma); scale bar: 500 μm. Adapted from: Allen Mouse Brain Connectivity Atlas (Oh et al., 2014), experiment 114155190. Website: © 2015 Allen Institute for Brain Science. Available from: http://connectivity.brain-map.org.

5-HT projections to the BLA arise predominantly from midbrain DRN neurons (Jacobs et al., 1978; Abrams et al., 2004), with only very sparse innervation from the median raphe nuclei (Jacobs et al., 1978; Vertes et al., 1999). 5-HT projections to the amygdaloid complex have been described in several ways, including the use of immunohistochemical labeling of either 5-HT (Steinbusch, 1981; Muller et al., 2007) or the 5-HTT (Sur et al., 1996). Both approaches reveal similar 5-HT innervation patterns in the amygdala of rodents. Typically, 5-HT axons project strongly to the BA, moderately to LA, basomedial and centromedial nuclei and only weakly to the centrolateral nucleus (Figure 3; Steinbusch, 1981; Sur et al., 1996; Muller et al., 2007). In the BLA, they innervate both PNs and INs (Muller et al., 2007).

To understand the effects of 5-HT on BLA microcircuits and fear learning, it is important to remember that LA and BA are not the same: they receive different inputs, project to different areas (reviewed in Sah et al., 2003; Duvarci and Pare, 2014), and are likely to be differentially modulated by 5-HT. It is also crucial to consider the localization of specific 5-HT receptor subtypes and defined cell types within the BLA microcircuits. For instance, a 5-HT receptor leading to membrane depolarization will have a profoundly different effect on network processing if it is located on a PN compared to an IN and *vice versa*.

### Glutamatergic Principal Neurons

Muller et al. (2007) found that 5-HT terminals form synaptic contacts with the distal dendrites of PNs. In the LA, both autoradiographic and *in situ* hybridization studies indicate high expression of 5-HT2C receptors (Li et al., 2003; Greenwood et al., 2012). As more than 80% of LA neurons are PNs, this is consistent with the 5-HT2C-mediated depolarizing effect of exogenous 5-HT found in LA PNs (Yamamoto et al., 2014). In contrast, in the BA McDonald and Mascagni (2007) reported labeling of PN dendrites obtained with two out of three 5-HT2A antibodies, while another antibody stained only PNs of the dorsolateral division of the LA. However, BA PNs are not depolarized by 5-HT2 agonists (Yamamoto et al., 2014). Thus, 5-HT2A receptors may not alter the membrane excitability in PNs somatically, but rather modulate the integration of dendritic inputs. Consistent with this idea, Chen et al. (2003) found that 5-HT2 activation enhanced NMDA receptor-mediated synaptic plasticity in putative PNs. In addition, a minority of PNs are directly hyperpolarized by 5-HT (Rainnie, 1999; Bocchio et al., 2015). This hyperpolarization could occur via activation of 5-HT1A receptors, which have been shown to be densely expressed in the BLA (Saha et al., 2010).

### GABAergic Interneurons

5-HT fibers target various IN classes in the BLA (Figure 2B; Muller et al., 2007; Bonn et al., 2013). Although PNs appear to be oppositely modulated by 5-HT in LA and BA, it is not clear yet whether IN populations express the same 5-HT receptors in both nuclei. Thus, most of the data reviewed in this section refer generally to the BLA, with no distinction between LA and BA.

The PV INs are believed to control fear learning via their regulation of PN spike timing and synchrony (Woodruff and Sah, 2007; Wolff et al., 2014) and their inhibition of SOM INs (Wolff et al., 2014). PV INs are innervated by 5-HT axons in the perisomatic region. This innervation is both synaptic and non-synaptic (Muller et al., 2007). In both LA and BA, PV INs express 5-HT2A receptors (McDonald and Mascagni, 2007; Jiang et al., 2009), which mediate membrane depolarization (Bocchio et al., 2015; Table 1 and Figure 1). Notably, mice genetically-engineered to overexpress the 5-HTT (5-HTTOE mice) have reduced 5-HT2A-mediated depolarization of PV INs (Bocchio et al., 2015; Figure 1D). 5-HTTOE mice exhibit low anxiety and impaired fear learning (Figure 1A; Jennings et al., 2006; Barkus et al., 2014; Line et al., 2014; Bocchio et al., 2015; McHugh et al., 2015), suggesting a link between reduced 5-HT2A receptor activation on PV INs and lower levels of anxiety/fear. In support of this, chronic citalopram administration, which is a clinically effective anxiolytic, also decreases forebrain 5-HT2A receptor binding (Günther et al., 2008).

The SOM INs, thought to contribute to fear learning via their dendritic inhibition of PNs (Wolff et al., 2014), are also targeted by 5-HT fibers (Muller et al., 2007). McDonald and Mascagni (2007) obtained labeling of ~30% of SOM INs using one out of three 5-HT2A antibodies. Thus, a minority of these neurons might also be depolarized via 5-HT2A signaling, but this has not been tested so far. Approximately 30% of SOM+ neurons co-express neuropeptide Y (NPY; McDonald, 1989). These NPY+ neurons receive dense 5-HT innervation (Bonn et al., 2013). Around 50–60% of NPY+ INs express 5-HT1A, suggesting they might be inhibited by 5-HT, while 30–40% express 5-HT2C, indicating they could be depolarized (Table 1, Bonn et al., 2013). Modulation of these neurons by 5-HT could be important for aspects of emotionality, because the activity of NPY cells has been proposed to be anxiolytic (Truitt et al., 2009). However, their involvement in fear memory processing is unclear.

In neocortex, prominent inhibition of SOM INs is provided by vasoactive intestinal peptide (VIP) INs, which promote disinhibition of pyramidal cells dendrites (Pfeffer et al., 2013). In the BLA, VIP INs have been shown to target PNs and calbindin (CB)-expressing INs (Muller et al., 2003). As most of BLA PV and SOM INs co-express CB, their inhibition by VIP INs is likely, but not yet demonstrated. VIP INs are innervated synaptically and non-synaptically by 5-HT axons (Muller et al., 2007), but it is not clear yet which 5-HT receptors they express. In neocortex, VIP INs are depolarized by 5-HT via ionotropic 5-HT3 receptors (Férézou et al., 2002), but virtually no VIP IN appears to express 5-HT3 receptors in the BLA (Mascagni and McDonald, 2007).

Together with VIP INs, “small” cholecystokinin (CCK) INs largely co-express calretinin (CR) in the BLA. In contrast, “large” CCK INs express CB but not VIP. In the HPC, CCK INs are a central target for 5-HT input and they express ionotropic 5-HT3 receptors (Morales and Bloom, 1997), which mediate fast synaptic depolarizations (Férézou et al., 2002; Varga et al., 2009). This seems to be the case in the BLA as well, but only a minority of large CCK INs could be labeled with a 5-HT3A antibody (Mascagni and McDonald, 2007). Surprisingly, the majority of 5-HT3A+ neurons do not belong to any previously characterized GABAergic population (Table 1, Mascagni and McDonald, 2007).

As well as postsynaptic innervation, axon terminals expressing 5-HT form appositions with 5-HT-negative axon terminals in the BLA (Muller et al., 2007). This suggests that 5-HT not only modulates BLA neuron excitability postsynaptically, but also the release of other neurotransmitters presynaptically. In agreement with this, pharmacological experiments performed in acute brain slices show that 5-HT can modulate glutamate release in the BLA (Cheng et al., 1998; Huang and Kandel, 2007), suggesting glutamatergic axon terminals might express 5-HT receptors. Taken together, these observations demonstrate that DRN 5-HT neurons innervate both BLA PNs and various types of GABAergic IN populations synaptically and extra-synaptically, and that 5-HT acts on both excitatory and inhibitory 5-HT receptors.

## 5-HT Modulation of BLA Information Processing

Having explored the complex localization of specific 5-HT receptors on distinct cell types, we now ask what are the functional consequences of 5-HT release on BLA circuit dynamics. To address this issue, we describe data originating from experiments involving electrophysiological recordings of BLA neurons and pharmacological activation/inhibition of 5-HT receptors.

### Lateral Amygdala

As previously emphasized, the LA is the main recipient of somatosensory information within the basolateral complex. The regulation of fear learning described in the previous sections could arise from 5-HT modulation of the inputs received by PNs and/or PN excitability. Perturbing these parameters could affect PN integration of CS and US information. *In vivo* recordings from LA neurons have shown that 5-HT depresses glutamatergic transmission from auditory cortex and thalamus (Stutzmann et al., 1998). *Ex vivo* patch-clamp studies have clarified that application of 5-HT depresses excitatory postsynaptic currents (EPSCs) from thalamic afferents in LA PNs via suppression of glutamate release mediated by 5-HT2 receptors (Yamamoto et al., 2012). As 5-HT2 receptors located on axon terminals are generally known to facilitate, and not depress, neurotransmitter release (Hasuo et al., 2002), this depression could occur via action of 5-HT on 5-HT2 receptors located on GABAergic INs (Stutzmann and LeDoux, 1999). At the same time, 5-HT application increases the excitability of LA PNs via 5-HT2C-mediated membrane depolarization (Yamamoto et al., 2014). Hence, 5-HT might bring PNs closer to the threshold for action potential generation and coincidently provide high-pass filtering (Abbott and Regehr, 2004) to suppress superfluous excitatory inputs from somatosensory areas. Together, these mechanisms might increase the signal-to-noise ratio for strong sensory stimuli and raise their chances of producing action potentials in LA PNs. To elucidate the mechanisms leading to altered fear learning upon 5-HT neurotransmission manipulation *in vivo*, it remains to be demonstrated whether 5-HT release modulates the induction of LTP at US and CS relaying synapses onto LA PNs.

### Basal Amygdala: Effects on Excitatory Transmission

The BA receives strong excitatory glutamatergic inputs from areas implicated in high-level polymodal sensory processing and memory, such as the entorhinal cortex (McDonald and Mascagni, 1997), HPC (Kishi et al., 2006) and mPFC (McDonald et al., 1996). Additionally, BA PNs receive excitatory inputs from LA PNs (Pitkänen et al., 1995). Given that the innervation of BA by somatosensory areas is minor (LeDoux et al., 1987; Shi and Cassell, 1998), LA PNs might relay information about the CS and US to BA neurons (Pitkänen et al., 1997).

The BA has a high density of 5-HT axons (Sur et al., 1996). Therefore, 5-HT could modulate the relay of CS and US information to BA PNs, ultimately shaping freezing responses via their excitatory output to the CeA. Intracellular recordings from BA PNs have shown that 5-HT promotes depression of EPSPs at synapses between LA PNs and BA PNs (Cheng et al., 1998; Yamamoto et al., 2012). Cheng et al. (1998) found that presynaptic 5-HT1A receptors mediate this effect, while Yamamoto et al. (2012) reported that 5-HT2 receptors are involved. The former finding suggests that 5-HT1A receptors are located on LA PNs axon terminals that synapse onto BA PNs and activation of these receptors depresses glutamate release. In contrast, the latter result points towards an involvement of GABAergic INs (see below), because presynaptic 5-HT2 receptors are excitatory and known to facilitate glutamate release (Hasuo et al., 2002). Furthermore, high concentrations of 5-HT (100–300 μM) caused only a transient depression of BA field EPSPs evoked by stimulation of the LA; this short-term depression was followed by LTP mediated by 5-HT4 receptor activation (Huang and Kandel, 2007). Thus, it needs to be clarified whether release of endogenous 5-HT produces short-term depression followed by long-lasting facilitation at LA-BA synapses, or only the former.

Additionally, theta burst stimulation of the external capsule, a fiber bundle bordering the BLA laterally, triggers only short term potentiation of EPSPs evoked in BA PNs from electrical stimulation of the external capsule (Chen et al., 2003). However, activation of postsynaptic 5-HT2A receptors promotes induction of LTP at these synapses via facilitated NMDA function (Chen et al., 2003). This mechanism could occur via 5-HT2A receptors detected on PNs distal dendrites (McDonald and Mascagni, 2007).

Thus, release of endogenous 5-HT likely modulates short-term and long-term synaptic plasticity of excitatory synapses to BA PNs. As the external capsule contains fibers from many brain regions, it is important to establish whether 5-HT promotes LTP at specific pathways (e.g., HPC or mPFC). Furthermore, the impact of this plasticity for learning still needs to be understood.

### Basal Amygdala: Effects on Inhibitory Transmission

A parallel action of 5-HT in the BA is depolarization of GABAergic INs via 5-HT2 and 5-HT3 receptors (Rainnie, 1999; Jiang et al., 2009; Bocchio et al., 2015), resulting in increased inhibition onto PNs. This effect appears to be driven primarily by 5-HT2A-mediated depolarization of PV INs (Jiang et al., 2009; Bocchio et al., 2015), which in turn causes inhibition of PNs (Rainnie, 1999; Bocchio et al., 2015). When 5-HT is physiologically released instead of bath applied, PV IN activation might not lead to prolonged membrane hyperpolarization, but rather influence the precision and synchrony of PNs firing (Woodruff and Sah, 2007; Ryan et al., 2012).

It is not known whether 5-HT excites or inhibits SOM INs. The answer to this question is crucial, because SOM INs control the integration of excitatory inputs in PN dendrites and their inhibition promotes fear learning (Wolff et al., 2014). McDonald and Mascagni (2007) detected 5-HT2A receptor expression on some SOM INs, which suggests that 5-HT depolarizes these cells. However, this evidence is not categorical as only one out of three antibodies labeled ~30% of SOM INs. Expression of 5-HT1A and/or 5-HT2C receptors on NPY+ INs, which are virtually all SOM+ in the rat (McDonald, 1989), was recently described (Bonn et al., 2013). However, electrophysiological recordings from identified SOM INs are necessary to test the effects of 5-HT on this important IN population. Although the innervation of VIP INs by 5-HT axons has been shown (Muller et al., 2007), the class of 5-HT receptor(s) expressed and the consequences of 5-HT transmission on these neurons is unclear.

In summary, current knowledge suggests that 5-HT in the BA enhances the perisomatic inhibition of PNs via excitation of PV INs. This likely shapes PN spike precision and synchrony (Woodruff and Sah, 2007; Ryan et al., 2012). However, it is uncertain whether 5-HT also depolarizes SOM INs, leading to inhibition of PN distal dendrites. In the absence of direct 5-HT excitation of SOM INs, 5-HT-mediated depolarization of PV INs (Wolff et al., 2014) or VIP INs could lead to inhibition of SOM INs. This would, in turn, cause disinhibition of PN dendrites, which has been shown to facilitate learning (Wolff et al., 2014).

It is important to note that circuit dynamics have largely been investigated using bath application or iontophoresis of 5-HT. It must be verified whether physiological release of 5-HT from DRN neurons recapitulates these effects, as bath application of drugs may activate more extrasynaptic receptors and lead to non-physiological effects (Unal et al., 2015). Additionally, in a behaving rodent, specific stimuli may selectively activate subpopulations of DRN 5-HT neurons, which in turn could target 5-HT release to the LA or the BA, or to specific neuronal populations within these nuclei.

## 5-HT Modulation of BLA Theta Oscillations

Given the prominent role of 5-HT in the recruitment of PV INs, a GABAergic population thought to synchronize large numbers of PNs (Courtin et al., 2014; Amilhon et al., 2015), a pertinent question is whether 5-HT affects neuronal synchrony in the BLA during fear conditioning. The synchronous activity of large numbers of neurons can be monitored via local field potential (LFP) recordings, which reveal oscillatory patterns in different frequency bands occurring during specific brain states and behaviors (Buzsáki and Draguhn, 2004). These extracellular voltage signals arise predominantly from synaptic activity, but also from action potentials or intrinsic membrane oscillations occurring synchronously in many neurons (Buzsáki et al., 2012).

Oscillations in the theta frequency range (4–12 Hz) occur in the BLA *in vivo* (Paré and Collins, 2000). This rhythmic network activity likely originates from synaptic inputs from areas interconnected with the BLA (such as the HPC or the mPFC; Pape and Pare, 2010) but also from intrinsic theta resonance of PN membranes (Paré et al., 1995; Pape and Driesang, 1998). Synchronized theta oscillations between BLA, mPFC and HPC increase during fear memory retrieval (Seidenbecher et al., 2003; Lesting et al., 2011; Likhtik et al., 2014) and consolidation (Popa et al., 2010). Simultaneous LFP and unit recordings suggest that theta epochs reflect an enhanced theta rhythmicity of the firing of BLA neurons (Paré and Collins, 2000). This synchronized firing is believed to facilitate communication between regions in response to aversive stimuli (Seidenbecher et al., 2003; Popa et al., 2010; Lesting et al., 2011). In line with this, BLA neurons fire phase-locked with hippocampal (Bienvenu et al., 2012; Mańko et al., 2012) and entorhinal theta (Paré and Gaudreau, 1996).

A positive modulation of fear-driven BLA theta oscillations by 5-HT is suggested by data from LFP recordings in 5-HTT mutant mice during fear conditioning. Namely, 5-HTTOE mice, which exhibit low extracellular 5-HT levels, show attenuated fear-evoked theta oscillations in the BLA during fear memory acquisition and expression (Figure 1B; Barkus et al., 2014). Conversely, 5-HTTKO mice, which have high extracellular 5-HT concentrations, display enhanced theta synchronization between the BLA and the mPFC (Narayanan et al., 2011). These studies indicate that 5-HT levels might positively correlate with BLA theta power and theta synchrony across the BLA-mPFC-HPC axis. This view is consistent with the finding that DRN 5-HT neurons show intrinsic theta rhythmic firing (Kocsis and Vertes, 1992).

In mPFC and HPC, PV INs can play an essential role in controlling theta oscillations (Courtin et al., 2014; Amilhon et al., 2015). In the BLA, classes of PV INs fire in phase with hippocampal theta rhythms (Bienvenu et al., 2012). Importantly, recruitment of BA PV INs during fear memory retrieval (measured by c-Fos expression) is reduced in 5-HTTOE mice (Figure 1C; Bocchio et al., 2015). This deficit might be caused by a weaker depolarizing action of 5-HT on PV INs (Bocchio et al., 2015) and may, at least in part, underlie impaired fear learning in these mice (Barkus et al., 2014; Line et al., 2014; Bocchio et al., 2015; McHugh et al., 2015; Figure 1). Taken together, these findings point towards a link between 5-HT and BLA theta oscillations, plausibly via 5-HT-mediated depolarization of PV INs and their synchronization of large numbers of PNs via perisomatic inhibition. Notably, the deficient 5-HT activation of PV INs in 5-HTTOE mice appears to derive not only from heightened 5-HTT function, but also from aberrant 5-HT2A signaling (Bocchio et al., 2015). Therefore, it cannot be ruled out that differential theta rhythmicity in 5-HTTKO and 5-HTTOE mice emerges from alterations in BLA circuits during development and/or from 5-HT receptor compensatory changes. Furthermore, changes in theta oscillations could be driven by altered rhythmic inputs from other structures interconnected with the BLA, such as the HPC or the mPFC. Hence, temporally selective and reversible manipulation of 5-HT neurotransmission should be employed in the future to test whether a causal link between 5-HT, BLA PV INs, BLA theta oscillations and fear exists.

## Conclusions and Future Directions

In summary, whole brain changes in extracellular 5-HT levels (SSRIs, tryptophan depletion, genetic variations in the 5-HTT) influence the acquisition (and likely the expression) of cued fear memories. Selective manipulation of BLA 5-HT neurotransmission with lesions and optogenetics demonstrate that 5-HT modulation of fear learning could be caused, at least in part, by actions on BLA neurons. Specifically, 5-HT could orchestrate the BLA microcircuitry in several ways. First, 5-HT could shape LTP induction at LA PN synapses and control the formation of PN ensembles encoding the CS. This could occur via alterations in glutamatergic transmission from sensory thalamus and cortex to LA PNs and via PN depolarization. Second, 5-HT facilitates LTP induction at BA PNs excitatory synapses from axons in the external capsule. Third, 5-HT could increase theta oscillations and BA PNs spike synchrony via recruitment of PV INs. These scenarios need to be validated with further empirical evidence. We propose that examining the effects of endogenous 5-HT release on discrete GABAergic IN classes and subcellular compartments of PNs is key to comprehend the complex effects of 5-HT on BLA circuits and fear learning. Notably, accumulating evidence suggests that the amygdala does not only encode aversion, but more generally affective significance (Morrison and Salzman, 2010; Gore et al., 2015; Namburi et al., 2015). Thus, besides aversive learning, 5-HT release in the BLA might influence other behaviors, for example reward processing.

It is essential to take into consideration that, in parallel to the putative effects on BLA circuits described above, midbrain 5-HT neurons could shape defensive responses through action on other brain regions. For instance, 5-HT modulates PNs and INs in HPC and mPFC (for review Puig and Gener, 2015), two regions interacting with the BLA in fear memory processing. Thus, 5-HT might alter HPC-BLA-mPFC interplay. Moreover, 5-HT could control fear responses via a downstream effect on the CeA, as recently CeA 5-HT2A+ neurons have been shown to provide a switch from learned freezing to innate freezing responses (Isosaka et al., 2015). However, the physiological actions of 5-HT on defined CeA cell types and circuit dynamics remain to be thoroughly investigated.

As previously mentioned, findings from rodent models of genetically altered 5-HT levels (5-HTTKO and 5-HTTOE) should be confirmed with reversible, spatially and temporally precise manipulations. This is because in these animal models 5-HT neurotransmission is altered during gestation, brain development and early-life experiences. Given the role of 5-HT in neuronal circuit formation (Daubert and Condron, 2010), emotional and physiological alterations could arise from abnormalities occurring during development (e.g., Gross et al., 2002), rather than from aberrant 5-HT neurotransmission during fear acquisition and expression.

At least three outstanding questions remain to be addressed. First, do DRN 5-HT neurons encode aversive signals during fear conditioning? Second, what DRN physiology is able to drive 5-HT release in the BLA? Third, does 5-HT release in the BLA promote synaptic plasticity and facilitate fear learning? Recent advances in bioengineering allow recording and manipulation of genetically defined neuron populations with high spatial and temporal resolution, providing valuable tools to answer these questions. Unambiguous identification of DRN 5-HT neurons in behaving mice can be achieved through Cre-dependent expression of opsins followed by combined optical stimulation and extracellular recordings, a method named “optogenetic tagging” (Kvitsiani et al., 2013; Roux et al., 2014). Alternatively, Cre-dependent expression of genetically encoded calcium sensors followed by deep brain imaging using an integrated microscope equipped with a microendoscope can be employed (Ghosh et al., 2011; Ziv et al., 2013). These approaches could provide valuable information on the activity of DRN 5-HT neurons during the CS and US with millisecond-timescale precision.

Furthermore, optogenetic excitation of 5-HT axons in the BLA during fear conditioning might disentangle the effect of 5-HT release during CS and US presentation. Finally, optical stimulation of 5-HT axons combined with recordings from defined BLA neuron populations could reveal the effects of endogenous 5-HT release on BLA circuit dynamics, and determine whether these recapitulate the effects obtained in pharmacological studies. This approach could also demonstrate whether DRN 5-HT neurons exert their action onto BLA circuits via co-release of glutamate, as reported in the HPC, ventral tegmental area and nucleus accumbens (Varga et al., 2009; Liu et al., 2014).

Answering these questions will advance our knowledge not only of the biological underpinnings of emotional behavior, but also of the mechanisms of 5-HT signaling that, presumably, are implicated in the therapeutic effects of SSRIs. Dissecting the modulation of limbic circuits by endogenous 5-HT could ultimately contribute to the design of more specific and effective treatments for psychiatric disorders in which these circuits are dysregulated.

## Author Contributions

MB wrote the main draft of the manuscript and prepared the figures and the tables. SBM, DMB, TS, MC also contributed to the text and commented all aspects of the manuscript.

## Funding and Disclosure

This work was supported by the Medical Research Council, UK (award U138197106) and a Wellcome Trust Senior Fellowship award (Grant No. 087736). TS is a recipient of collaborative research grants from Lundbeck. DMB is a member of Lilly UK’s Centre of Cognitive Neuroscience.

## Conflict of Interest Statement

The authors declare that the research was conducted in the absence of any commercial or financial relationships that could be construed as a potential conflict of interest.
